# Supplementary material for: EEG-Based Neurofeedback in Athletes and Non-Athletes: A Scoping Review of Outcomes and Methodologies
Source: Bioengineering (Basel). 2025 Nov 3;12(11):1202. doi: 10.3390/bioengineering12111202 (PMC12650374; doi:10.3390/bioengineering12111202)
Supplement: Supplementary file 1 [file bioengineering-12-01202-s001.zip › bioengineering-3865423-supplementary.pdf]

EEG-Neurofeedback in Athletes and Non-Athletes: A Scoping Review of Outcomes, Methodological Rigor, and Reproducibility Gaps

Rui Manuel Guerreiro Zacarias[1] (https://orcid.org/0000-0002-5542.9600, rmzacarias@ualg.pt),

Thejani Bulathwatta[2] (https://orcid.org/0009-0006-3165-4607, thejani.bulathwatta@ug.edu.pl),

Ilona Bidzan-Bluma[2] (https://orcid.org/0000-0002-0654-2068, ilona.bidzan@ug.edu.pl),

Saúl Neves de Jesus[1] (https://orcid.org/0000-0003-2019-1011, snjesus@ualg.pt),

João Correia[1] (https://orcid.org/0000-0001-6624-7012, jcorreia@ualg.pt)

Affiliations

[1] University of Algarve, Faculty of Human and Social Sciences, Faro, Portugal

[2] University of Gdańsk, Institute of Psychology, Gdańsk, Poland

Correspondence to:

Rui Manuel Guerreiro Zacarias

University of Algarve, Faculty of Human and Social Sciences Campus de Gambelas, 8005-139 Faro, Portugal

Email: rmzacarias@ualg.pt

Telephone: +351 289 800100/900

Appendix S1. Preferred Reporting Items for Systematic reviews and Meta-Analyses extension for Scoping Reviews (PRISMA-ScR) Checklist.

Section	Item	PRISMA-ScR Checklist Item	Reported on Page #
TITLE	1	Identify the report as a scoping review.	1
ABSTRACT	2	Provide a structured summary (background, objectives, eligibility criteria, sources, charting methods, results, conclusions).	1-2
INTRODUCTION	3	Describe rationale in context of what is known.	3
INTRODUCTION	4	State explicit objectives and review questions.	3-4
METHODS	5	Indicate whether a protocol exists and where accessed (e.g., OSF).	4 (OSF DOI: https://doi.org/10.17605/OSF.IO/XCUWY)
METHODS	6	Specify eligibility criteria (years, language, publication status, rationale).	4-5
METHODS	7	Describe all information sources (databases, coverage, search date).	5
METHODS	8	Present full electronic search strategy for at least 1 database.	Appendix A
METHODS	9	State process for selecting sources of evidence (screening, eligibility).	5–6 (Rayyan, PRISMA flow diagram)

METHODS	10	Describe data charting process (calibrated forms, duplicate extraction).	6
METHODS	11	List and define all variables sought.	6-7
METHODS	12	Critical appraisal of sources of evidence, if done.	Not applicable
METHODS	13	Methods for handling and summarizing charted data.	7
RESULTS	14	Numbers screened, excluded, included (flow diagram).	8 (Figure 1)
RESULTS	15	Characteristics of included studies.	8–13 (Tables 1A–1C)
RESULTS	16	Critical appraisal within sources of evidence.	Not applicable
RESULTS	17	Relevant data from each included source.	10–13 (Tables 1A–1C)
RESULTS	18	Summarize charting results in relation to questions.	13-23
DISCUSSION	19	Summarize main results, link to objectives, relevance to key groups.	24-26
DISCUSSION	20	Limitations of the scoping review process.	27
DISCUSSION	21	Interpretation, implications, next steps.	27-28
FUNDING	22	Sources of funding and role of funders.	29

Notes:

JBI = Joanna Briggs Institute; PRISMA-ScR = Preferred Reporting Items for Systematic Reviews and Meta-Analyses extension for Scoping Reviews.

* *Where sources of evidence are compiled from (e.g., bibliographic databases, social media platforms, websites).*

† *A more inclusive/heterogeneous term used to account for the different types of evidence or data sources that may be eligible in a scoping review as opposed to only studies.*

‡ *Refers to the process of data extraction in a scoping review (Arksey & O'Malley framework; Levac et al.; JBI guidance).*

§ *Process of examining research evidence to assess its validity, results, and relevance before using it to inform decisions. This is used for Items 12 and 19 instead of "risk of bias".*

Reference:

Tricco, A. C., Lillie, E., Zarin, W., O'Brien, K. K., Colquhoun, H., Levac, D., Moher, D., Peters, M. D. J., Horsley, T., Weeks, L., Hempel, S., Akl, E. A., Chang, C., McGowan, J., Stewart, L., Hartling, L., Aldcroft, A., Wilson, M. G., Garritty, C., … Straus, S. E. (2018). PRISMA extension for scoping reviews (PRISMA-ScR): Checklist and explanation. *Annals of Internal Medicine, 169*(7), 467–473. https://doi.org/10.7326/M18-0850

Appendix S2. Search strategy used for each of the Databases.

Database	Search strategy	Results Apr 25th 2025
**Medline / PubMed**	("Neurofeedback"[MeSH Terms] OR "neurofeedback*"[Text Word] OR "neuro feedback*"[Text Word] OR "alpha feedback*"[Text Word] OR "eeg feedback*"[Text Word] OR "Electromyography Feedback"[Text Word] OR "biofeedback, psychology"[MeSH Terms] OR "biofeedback*"[Text Word] OR "Psychology Biofeedback"[Text Word] OR "physiological feedback*"[Text Word]) AND ("neurophysiological marker*"[Text Word] OR "neurophysiologic marker*"[Text Word] OR "ERP"[Title/Abstract] OR "qEEG"[Title/Abstract] OR "LORETA"[Title/Abstract] OR ("Cognition"[MeSH Terms] OR "cognition*"[Text Word] OR "Cognitive"[Text Word])) AND ("Athletes"[MeSH Terms] OR "athlete*"[Text Word] OR "Sports"[MeSH Terms] OR "sport*"[Text Word] OR "athletic*"[Text Word] OR "Exercise"[MeSH Terms] OR "exercise*"[Text Word] OR "physical activit*"[Text Word])	329
**Embase**	('neurofeedback'/exp OR 'neurofeedback*':ti,ab,kw,de,dn,df,mn,tn OR 'neuro feedback*':ti,ab,kw,de,dn,df,mn,tn OR 'alpha feedback*':ti,ab,kw,de,dn,df,mn,tn OR 'eeg feedback*':ti,ab,kw,de,dn,df,mn,tn OR 'electromyography feedback':ti,ab,kw,de,dn,df,mn,tn OR 'biofeedback'/exp OR 'biofeedback*':ti,ab,kw,de,dn,df,mn,tn OR 'psychology biofeedback':ti,ab,kw,de,dn,df,mn,tn OR 'physiological feedback*':ti,ab,kw,de,dn,df,mn,tn) AND ('neurophysiological marker*':ti,ab,kw,de,dn,df,mn,tn OR 'neurophysiologic marker*':ti,ab,kw,de,dn,df,mn,tn OR 'erp':ti,ab,kw OR 'qeeg':ti,ab,kw OR 'loreta':ti,ab,kw OR 'cognition'/exp OR 'cognition*':ti,ab,kw,de,dn,df,mn,tn OR 'cognitive':ti,ab,kw,de,dn,df,mn,tn) AND ('athlete'/exp OR 'athlete*':ti,ab,kw,de,dn,df,mn,tn OR 'sport'/exp OR 'sport*':ti,ab,kw,de,dn,df,mn,tn OR 'athletic*':ti,ab,kw,de,dn,df,mn,tn OR 'exercise'/exp OR 'exercise*':ti,ab,kw,de,dn,df,mn,tn OR 'physical activit*':ti,ab,kw,de,dn,df,mn,tn)	1,558
**Scopus**	TITLE-ABS-KEY(neurofeedback* OR "neuro feedback*" OR "alpha feedback*" OR "eeg feedback*" OR "Electromyography Feedback" OR biofeedback* OR "physiological feedback*" OR "Brainwave Feedback*" OR "Psychophysiologic Feedback*") AND TITLE-ABS-KEY("neurophysiological marker*" OR "neurophysiologic marker*" OR "ERP" OR "qEEG" OR "LORETA" OR cognition* OR Cognitive) AND TITLE-ABS-KEY(athlete* OR sport* OR athletic* OR exercise* OR "physical activit*")	647
**Web of Science**	TS=(neurofeedback* OR "neuro feedback*" OR "alpha feedback*" OR "eeg feedback*" OR "Electromyography Feedback" OR biofeedback* OR "physiological feedback*" OR "Brainwave Feedback*" OR "Psychophysiologic Feedback*") AND TS=("neurophysiological marker*" OR "neurophysiologic marker*" OR "ERP" OR "qEEG" OR "LORETA" OR cognition* OR Cognitive) AND TS=(athlete* OR sport* OR athletic* OR exercise* OR "physical activit*")	371
**PsycINFO**	((Title: neurofeedback* OR Title: "neuro feedback*" OR Title: "alpha feedback*" OR Title: "eeg feedback*" OR Title: "Electromyography Feedback" OR Title: biofeedback* OR Title: "physiological feedback*" OR Title: "Brainwave Feedback*" OR Title: "Psychophysiologic Feedback*") AND (Title: "neurophysiological marker*" OR Title: "neurophysiologic marker*" OR Title: "ERP" OR Title: "qEEG" OR Title: "LORETA" OR Title: cognition* OR Title: Cognitive) AND (Title: athlete* OR Title: sport* OR Title: athletic* OR Title: exercise* OR Title: "physical activit*")) OR ((Abstract: neurofeedback* OR Abstract: "neuro feedback*" OR Abstract: "alpha feedback*" OR Abstract: "eeg feedback*" OR Abstract: "Electromyography Feedback" OR Abstract: biofeedback* OR Abstract: "physiological feedback*" OR Abstract: "Brainwave Feedback*" OR Abstract: "Psychophysiologic Feedback*") AND (Abstract: "neurophysiological marker*" OR Abstract: "neurophysiologic marker*" OR Abstract: "ERP" OR Abstract: "qEEG" OR Abstract: "LORETA" OR Abstract: cognition* OR Abstract: Cognitive) AND (Abstract: athlete* OR Abstract: sport* OR Abstract: athletic* OR Abstract: exercise* OR Abstract: "physical activit*")) OR ((Keywords: neurofeedback* OR Keywords: "neuro feedback*" OR Keywords: "alpha feedback*" OR Keywords: "eeg feedback*" OR Keywords: "Electromyography Feedback" OR Keywords: biofeedback* OR Keywords: "physiological feedback*" OR Keywords: "Brainwave Feedback*" OR Keywords: "Psychophysiologic Feedback*") AND	175

	(Keywords: "neurophysiological marker*" OR Keywords: "neurophysiologic marker*" OR Keywords: "ERP" OR Keywords: "qEEG" OR Keywords: "LORETA" OR Keywords: cognition* OR Keywords: Cognitive) AND (Keywords: athlete* OR Keywords: sport* OR Keywords: athletic* OR Keywords: exercise* OR Keywords: "physical activit*"))	
**Cochrane Library**	([mh Neurofeedback] OR neurofeedback*:ti,ab,kw OR ("neuro" NEXT feedback*):ti,ab,kw OR ("alpha" NEXT feedback*):ti,ab,kw OR ("eeg" NEXT feedback*):ti,ab,kw OR "Electromyography Feedback":ti,ab,kw OR [mh "biofeedback, psychology"] OR biofeedback*:ti,ab,kw OR "Psychology Biofeedback":ti,ab,kw OR ("physiological" NEXT feedback*):ti,ab,kw OR ("Brainwave" NEXT Feedback*):ti,ab,kw OR ("Psychophysiologic" NEXT Feedback*):ti,ab,kw) AND (("neurophysiological" NEXT marker*):ti,ab,kw OR ("neurophysiologic" NEXT marker*):ti,ab,kw OR ERP:ti,ab,kw OR qEEG:ti,ab,kw OR LORETA:ti,ab,kw OR [mh Cognition] OR cognition*:ti,ab,kw OR Cognitive:ti,ab,kw) AND ([mh Athletes] OR athlete*:ti,ab,kw OR [mh Sports] OR sport*:ti,ab,kw OR athletic*:ti,ab,kw OR [mh Exercise] OR exercise*:ti,ab,kw OR ("physical" NEXT activit*):ti,ab,kw)	198
**LILACS**	(neurofeedback* OR "neuro feedback" OR "alpha feedback" OR "eeg feedback" OR "Electromyography Feedback" OR biofeedback* OR "physiological feedback" OR "Brainwave Feedback" OR "Psychophysiologic Feedback" OR "Brainwave Biofeedbacks" OR "Alpha Biofeedbacks" OR "Alpha Feedbacks" OR "Brainwave Feedbacks" OR "EEG Feedbacks" OR "Psychology Biofeedbacks" OR "Physiological Feedbacks" OR neurorretroalimentação OR biorretroalimentação OR "Feedback Alfa" OR "Feedback de Onda Cerebral" OR retroalimentação OR neurorretroalimentación OR biorretroalimentación OR retroalimentación) AND ("neurophysiological marker" OR "neurophysiologic marker" OR "neurophysiological markers" OR "neurophysiologic markers" OR "ERP" OR "qEEG" OR "LORETA" OR cognition* OR cognitive OR "marcador neurofisiológico" OR "marcadores neurofisiológicos" OR cognição OR cognitiva OR cognitivo OR cognición) AND (athlete* OR sport* OR athletic* OR exercise* OR "Physical Activity" OR "Physical Activities" OR atletas OR atleta OR deportistas OR deportista OR esportes OR esporte OR esportivas OR esportiva OR esportivos OR esportivo OR desportes OR desportos OR deportes OR "actividades deportivas" OR exercício OR exercícios OR "Atividade Física" OR "Atividades Físicas" OR "Práticas Corporais" OR "Treinamento Físico" OR ejercicio OR "Actividad Física" OR "Entrenamiento Físico" OR "Prácticas Corporales") AND db:("LILACS") AND instance:"regional"	18
**ProQuest Dissertations & Theses Citation Index**	TS=(neurofeedback* OR "neuro feedback*" OR "alpha feedback*" OR "eeg feedback*" OR "Electromyography Feedback" OR biofeedback* OR "physiological feedback*" OR "Brainwave Feedback*" OR "Psychophysiologic Feedback*") AND TS=("neurophysiological marker*" OR "neurophysiologic marker*" OR "ERP" OR "qEEG" OR "LORETA" OR cognition* OR Cognitive) AND TS=(athlete* OR sport* OR athletic* OR exercise* OR "physical activit*")	52
**Tripdatabase**	(Neurofeedback OR "neuro feedback") AND (Athlete* OR sport* OR exercise*)	68
**Google Scholar**	(neurofeedback OR Neurofeedbacks OR "neuro feedback" OR "alpha feedback" OR "eeg feedback" OR "Electromyography Feedback" OR biofeedback OR Biofeedbacks OR "physiological feedback" OR "Brainwave Feedback" OR "Psychophysiologic Feedback" OR "Alpha Feedbacks" OR "Brainwave Feedbacks" OR "EEG Feedbacks" OR "Psychology Biofeedbacks" OR "Physiological Feedbacks" OR Neurorretroalimentação OR Biorretroalimentação OR "Feedback Alfa" OR "Feedback de Onda Cerebral" OR Retroalimentação OR Neurorretroalimentación OR Biorretroalimentación OR Retroalimentación) AND (athlete OR sport OR athletic OR exercise OR Athletes OR Athletics OR sports OR Exercises OR "Physical Activity" OR "Physical Activities" OR Atletas OR Atleta OR deportistas OR deportista OR Esportes OR Esporte OR Esportivas OR Esportiva OR Esportivos OR Esportivo OR Desportes OR Desportos OR Deportes OR "actividades deportivas" OR Exercício OR Exercícios OR "Atividade Física" OR "Atividades Físicas" OR "Práticas Corporais" OR "Treinamento Físico" OR Ejercicio OR "Actividad Física" OR "Entrenamiento Físico" OR "Prácticas Corporales") AND ("neurophysiological marker" OR "neurophysiologic marker" OR "neurophysiological markers" OR "neurophysiologic markers" OR "ERP" OR "qEEG" OR "LORETA" OR cognition OR Cognitive OR "marcador neurofisiológico" OR "marcadores neurofisiológicos" OR Cognição OR Cognitiva OR Cognitivo OR Cognición)	100

Search strategies were performed for each database by using specifics words combinations and truncations with the support of a librarian.

Appendix S3. Excluded articles and reasons for exclusion (n = 22).

Study	Reason for exclusion	Justification
Gruzelier et al., 2006	3	Relied mainly on subjective outcomes, lacking objective neurophysiological/performance measures.
Isrctn, 2008	1	Clinical population (stroke patients), not within the scope of non-clinical/athletic samples.
Rozengurt et al., 2009	2	Insufficient methodological detail; single session design, focused mainly on sleep/memory consolidation rather than structured neurofeedback training outcomes in athletes.
Vernon et al., 2009	6	Systematic/narrative review; not an original experimental study with primary data.
Linden et al., 2010	6	Narrative review/panel discussion; lacks primary empirical data or detailed experimental methodology.
Rostami et al., 2010	7	Duplicate record; same study retrieved with inconsistent metadata (different year and journal).
Rozengurt et al., 2011	3	Relied primarily on subjective outcomes (self-reports, procedural motor learning), lacking strong objective performance validation.
Kovaleva et al., 2012	3	Relied primarily on subjective outcomes (e.g., Spielberger anxiety scale, self-reports), limiting objective neurophysiological evidence.
Rogala et al., 2016	6	This is a narrative/systematic review, not a primary empirical study.
Ims, 2017	1	Clinical population (traumatic brain injury), which falls outside the scope of the review.

Mirifar et al., 2017	6	Systematic review, not an original empirical study, therefore outside the inclusion criteria.
Thibault, Lifshitz & Raz, 2017	4	Commentary/opinion article, not an original empirical study, therefore excluded.
Thibault & Raz, 2017	4	Opinion/theoretical article, not presenting original empirical data, excluded.
Xiang et al., 2018	6	Systematic review/meta-analysis; not original empirical research, therefore excluded.
Actrn, 2018	1	Clinical population (PTSD in adult refugees); not within the scope of this review.
Gong et al., 2021	6	Systematic/narrative review, not an original empirical study, therefore excluded.
Brito et al., 2022	6	Systematic review and meta-analysis, not an original empirical study, therefore excluded.
Nct, 2023	5	Protocol/clinical trial registration only, full text and study results unavailable, therefore excluded.
Tosti et al., 2024	6	Systematic review and meta-analysis, not an original empirical study, therefore excluded.
Wu et al., 2024	7	Duplicate record with inconsistent metadata (year mismatch across databases: 2023/2024), therefore excluded.
Dana, Rafiee, & Gholami, 2019	1	Study includes a juvenile population in a clinical developmental context, which is not within the scope of the review.
Mikicin & Orzechowski, 2018	1	Study conducted with a specific population (military/police shooters), not considered within the target definition of athletes for this review.

Reasons for Exclusion

1. Clinical populations (e.g., neurological or psychiatric diagnoses).
2. Insufficient methodological detail on the neurofeedback protocol or outcomes.
3. Exclusively subjective outcomes (e.g., self-perceived performance).
4. Non–peer-reviewed publications (e.g., conference abstracts, opinion articles, technical reports).
5. Full text unavailable or no response from corresponding authors after three attempts.
6. Systematic reviews, narrative reviews, and meta-analyses.
7. Duplicate record / incorrect metadata (same study retrieved more than once, with inconsistent year, journal, or database indexing errors).
